# Endoscopic full thickness resection for early colon cancer in Lynch syndrome

**DOI:** 10.1007/s10689-019-00132-w

**Published:** 2019-05-20

**Authors:** Alexandra M. J. Langers, Jurjen J. Boonstra, James C. H. Hardwick, Jolein van der Kraan, Arantza Farina Sarasqueta, Hans F.A. Vasen

**Affiliations:** 1 Department of Gastroenterology and Hepatology, Leiden University Medical Centre, PO Box 9600, 2300 RC Leiden, The Netherlands; 2 Department of Pathology, Leiden University Medical Centre, Leiden, The Netherlands

**Keywords:** Lynch syndrome, eFTR, Endoscopic resection, Full-thickness resection, Colorectal cancer

## Abstract

Subtotal colectomy is usually the therapy of choice in Lynch syndrome patients diagnosed with colon cancer. In patients who develop cancer after the age of 50–60 years, segmental colectomy is considered a good alternative. Although the endoscopic treatment of early colorectal cancer in non-Lynch patients has increased in the last decades, almost all patients with a Lynch syndrome-associated colorectal malignancy undergo surgery, even if the tumour is diagnosed in a (very) early stage. One of the endoscopic treatment options for early colorectal cancer is an endoscopic full thickness resection (eFTR). This treatment modality allows optimal pathological examination of the resection specimen, as a transmural resection is performed with optimal T-staging of the tumour. We report a case of a 62 year old man, diagnosed with MSH2-Lynch syndrome, who underwent successful eFTR treatment of an early (pT1) colon cancer located in the ascending colon, with no signs of recurrence 12 months after treatment. We discuss the pros and cons of endoscopic resection of early colorectal carcinoma in Lynch syndrome patients.

## Introduction

Lynch syndrome (LS) patients undergo regular endoscopic surveillance because of their increased risk of colorectal cancer (CRC). The aim of this surveillance is to either prevent colorectal cancer by removal of a precursor lesion, or detection of a colorectal cancer that can be treated with curative intent [1]. Usually, LS patients who are diagnosed with colorectal cancer undergo a subtotal or segmental colorectal resection. The options for endoscopic treatment of early colorectal cancer have increased substantially in the last decade. Endoscopic full-thickness resection (eFTR), is a novel treatment option for early colorectal cancer. We report the first case of a LS patient who underwent eFTR treatment for an early colorectal carcinoma located in the ascending colon, and discuss the advantages and possible disadvantages of using this technique in LS patients.

## Case

**Fig. 1:**
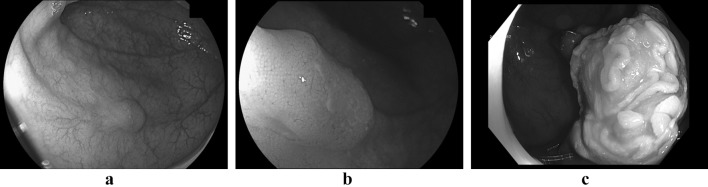
**a** white light image (overview) of the early CRC with central ulceration. **b** Close-up of the early cancer. **c** Local situation after resection of the lesion with the full thickness resection device; Over-The-Scope-Clip in good position

**Fig. 2:**
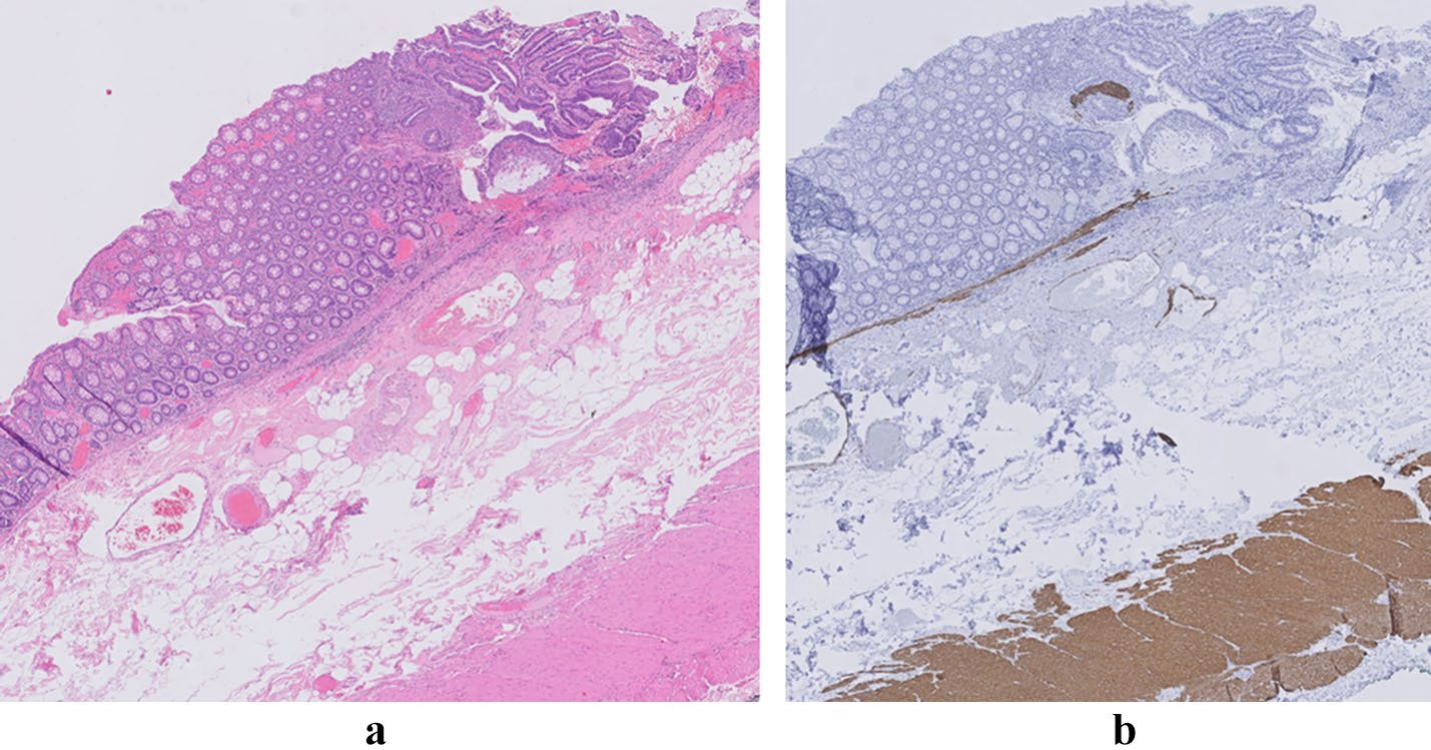
**a** H&E stain of the resection specimen. **b** Desmin stain of the resection specimen

A 62 years old man had been referred to our outpatient clinic because of recently diagnosed Lynch syndrome due to an MSH2 mutation. His family history was negative for any kind of cancer. He had been treated with curative intent for pancreatic cancer four years ago. The cancer was located in the pancreatic tail and histology showed a poorly differentiated adenocarcinoma of the pancreaticobiliary type, 6 centimetres in size, that extended into the spleen. The tumour could be radically resected; there were no positive lymph nodes. Two years later, he underwent a left-sided nephrectomy because of a low grade (grade I) urothelial cell carcinoma of the pyelum of the left kidney. Both malignancies showed loss of expression of MSH2, and subsequent genetic testing revealed a germ line mutation in the MSH2 gene (c.2090G>A p.Cys697Tyr in exon 13). In a functional test, this missense mutation shows mismatch repair deficiency and is therefore classified as a pathogenic mutation [2]. At his index colonoscopy, a small but suspect lesion was found in the ascending colon. There was a slight ulceration of the surface of a 7 × 7 mm Paris Is lesion and careful inspection using a Fujinon^®^ Slim zoom video colonoscope (Eluxeo 700 series; 135 × maximum magnification) showed a Kudo Vn pit pattern, suggestive of an early invasive cancer (Fig. 1a, b). The colonoscopy was aborted and the different therapeutic options, as well as the pros and cons of each option, were discussed with the patient and his son. Besides the possibility of a segmental colectomy or subtotal colectomy, we also discussed the option of removing the lesion by eFTR. The patient consented with the option of endoscopic en bloc removal of the lesion and a colonoscopy under propofol sedation was scheduled to remove the lesion endoscopically. This procedure was carried out as follows: first the margins of the lesion were marked with a marking probe. Then the colonoscope was withdrawn and the Full-Thickness Resection Device (FTRD, Ovesco^®^) was mounted on the colonoscope. The colonoscope was re-inserted into the caecum and an FTRD^®^ Grasper was used to draw the lesion into the cap of the eFTR system. When all circumferential markings were visible inside the cap, the over-the-scope clip (OTSC) was released and immediately afterwards the tissue within the OTSC was resected using the pre-mounted snare within the cap and the pure cut setting of the Erbe^®^ coagulation system. The endoscope was withdrawn with the specimen in the cap and the specimen was subsequently pinned on a cork board for optimal pathological evaluation. After re-introducing the endoscope a nice full-thickness wound was seen with the OTSC in good position (Fig. 1c). Pathological examination showed a pT1 moderate to well-differentiated adenocarcinoma of the ascending colon with invasion into the submucosa, Kikuchi level sm1, with an invasive component of 0.3 cm, no lymphovascular invasion and a free resection margin of at least 2 mm (Fig. 2a H&E, Fig. 2b desmin immunohistochemistry). There was grade I tumour budding and loss of MSH2 staining. After discussion in the multidisciplinary team and shared decision making with the patient, we agreed not to opt for additional surgical resection, but for close follow-up by regular colonoscopy. Colonoscopy 12 months after the procedure showed no sign of residual or recurrent cancer and a CT scan, that was carried out in the follow-up of his urothelial call carcinoma, showed no sign of distant metastases 12 months after the endoscopic resection.

## Discussion

The introduction of population screening for CRC has led to a large increase in the detection of early colorectal cancer in the general population. The options for endoscopic removal of these early cancers have increased in the last decades. Endoscopic removal of early CRC with an *en bloc* endoscopic mucosal resection (EMR), endoscopic submucosal dissection (ESD) or eFTR can be curative, without the need for additional surgery. These endoscopic techniques have a lower morbidity and mortality than colorectal surgery, where 30-day mortality rates of 2.4% and a 90-day readmission rates of 21.8% have been reported [3]. Furthermore, long term complications like adhesive small bowel obstruction occur in a substantial number of patients after partial colectomy [4].

Both ESD and eFTR are suitable techniques for the treatment of early colorectal cancers, invading into the submucosa layer but not beyond it. Although intra- or postprocedural bleeding and perforation do occur in ESD and eFTR treatment, the number of patients requiring surgery is low and mortality after endoscopic resection is rare [5, 6]. The introduction of endoscopic submucosal dissection (ESD) has made it possible to remove sessile lesions of any diameter including those with a suspicion of early invasion. For smaller lesions with suspicion of malignancy located anywhere in the colon, eFTR is a new therapeutic modality. Because of the limited size of the cap of the eFTR device, the maximum size of the lesion should not exceed 3 cm. Not only the diameter, but also the shape of the lesion should be taken into account and in case of a more voluminous lesion the maximum diameter is probably 2–2.5 cm or even smaller. An advantage of the eFTR technique is that a full thickness specimen can be obtained, which allows the pathologist to perform optimal T-staging of the tumour in case of malignancy. In non-LS patients, eFTR is considered to be a feasible and safe technique for the treatment for early (and small) CRC [6].

Because of the regular endoscopic surveillance, early cancer is detected more frequently in LS patients than in the general population. A recent analysis of 78 incident CRCs in LS patients demonstrated that the median tumour diameter was 2.5 cm and lymph node metastases were absent in 83% of the cases [7], suggesting that part of these cancers may be suitable for eFTR treatment. However, the guideline of the American College of Surgery (ACG) recommends colectomy with ileorectal anastomosis (IRA) as the preferred treatment of young (below 60–65 years of age) LS patients with colon cancer and in patients with benign polyps that cannot be removed endoscopically. Segmental colectomy is mentioned as an alternative in LS patients unsuitable for total colectomy [8]. One of the main reasons for this extensive surgery is the lifetime risk of a second CRC, a risk that is particularly high in younger CRC patients. De Vos tot Nederveen Cappel et al. compared the overall life expectancy gain of subtotal colectomy versus segmental resection in LS patients with colorectal cancer, and concluded that at the age of 27 and 47 years this gain was 2.3 and 1 years respectively; at the age of 60, the gain of life expectancy was only 4 months [9]. In younger patients, the life expectancy increase is substantial, and therefore a subtotal colectomy is still considered the therapy of choice in patients under 50–60 years of age. In older patients, the average gain in life expectancy is much smaller, whereas the risk of surgery increases, especially in patients with cardiopulmonary comorbidity.

A possible disadvantage of endoscopic resection of malignant polyps, compared to surgical resection, is the inability to define the N-status, as no lymph nodes are removed by this technique. After endoscopic removal of a malignant polyp, the risk of lymph node metastases can be estimated based on tumour characteristics such as differentiation grade (good/moderate or poor), lymphatic or vascular invasion and the presence or absence of a positive resection margin [10]. In the Japanese guidelines, additional risk factors such as tumour budding and the depth of submucosal invasion are also taken into account [11]. When the estimated risk of lymph node metastases is low, additional surgical resection with lymph node dissection is not recommended and close follow-up is advised, as was performed in our patient. Compared to sporadic CRC, the incidence of lymph node metastases in LS patient with early CRC is very low [7]. Therefore, the use of the algorithm to estimate the risk of lymph node positivity appears to be safe in LS patients and might even overestimate the need for additional surgical resection in these patients.

Taking into account the risks and benefits of colorectal surgery, we believe that subtotal (or partial) colectomy is still the therapy of choice in young LS patients diagnosed with CRC. However, we also believe that the option of endoscopic resection should be discussed with LS patients with an endoscopically resectable early colorectal cancer, and this discussion should be part of the process of shared decision making. In this specific case, the patient was over 60 years of age, had been treated for two malignancies with unclear prognosis within the 5 years preceding his colorectal cancer and had a low-risk T1 colorectal cancer; these factors all contributed to the decision to consider endoscopic treatment and refrain from additional surgery. More robust and long term data about this procedure are needed to determine the exact position of eFTR in LS patients and the decision to treat early cancers in LS patients by eFTR should be taken with caution. Nevertheless, especially in older LS patients, in patients with severe comorbidity and in patients who refuse major surgery, endoscopic resection should be considered as an alternative to surgical resection for the treatment of early colorectal cancer.
